# A Metabolomic Approach to Clarifying the Effect of AST-120 on 5/6 Nephrectomized Rats by Capillary Electrophoresis with Mass Spectrometry (CE-MS)

**DOI:** 10.3390/toxins4111309

**Published:** 2012-11-14

**Authors:** Yasutoshi Akiyama, Yoichi Takeuchi, Koichi Kikuchi, Eikan Mishima, Yasuaki Yamamoto, Chitose Suzuki, Takafumi Toyohara, Takehiro Suzuki, Atsushi Hozawa, Sadayoshi Ito, Tomoyoshi Soga, Takaaki Abe

**Affiliations:** 1 Department of Community Health Promotion, Tohoku University Graduate School of Medicine, Sendai 980-8574, Japan; Email: y-akiyama@med.tohoku.ac.jp; 2 Division of Nephrology, Endocrinology and Vascular Medicine, Tohoku University Graduate School of Medicine, Sendai 980-8574, Japan; Email: muu-tko@med.tohoku.ac.jp (Y.T.); koichikikuchithk@yahoo.co.jp (K.K.); eikan@med.tohoku.ac.jp (E.M.); drow_rorrim@yahoo.co.jp (Y.Y.); chitose@med.tohoku.ac.jp (C.S.); toyohara@med.tohoku.ac.jp (T.T.); suzuki2i@med.tohoku.ac.jp (T.S.); db554@med.tohoku.ac.jp (S.I.); 3 Department of Clinical Biology and Hormonal Regulation, Tohoku University Graduate School of Medicine, Sendai 980-8574, Japan; 4 Department of Preventive Medicine and Epidemiology, Tohoku Medical Megabank Organization, Tohoku University, Sendai 980-8573, Japan; Email: hozawa-thk@umin.ac.jp; 5 Institute for Advanced Biosciences, Keio University, Tsuruoka, Yamagata 997-0052, Japan; Email: soga@sfc.keio.ac.jp; 6 Division of Medical Science, Tohoku University Graduate School of Biomedical Engineering, Sendai 980-8574, Japan

**Keywords:** AST-120, uremic toxin, CE-MS

## Abstract

The oral adsorbent AST-120 is composed of spherical carbon particles and has an adsorption ability for certain small-molecular-weight compounds that accumulate in patients with chronic kidney disease (CKD). So far, very few compounds are known to be adsorbed by AST-120 *in vivo*. To examine the effect of AST-120 *in vivo*, we comprehensively evaluated the plasma concentrations of 146 compounds (61 anions and 85 cations) in CKD model rats, with or without four weeks of treatment with AST-120. By capillary electrophoresis with mass spectrometry, we identified 6 anions and 17 cations that were significantly decreased by AST-120 treatment. In contrast, we also identified 2 cations that were significantly increased by AST-120. Among them, 4 anions, apart from indoxyl sulfate and hippurate, and 19 cations were newly identified in this study. The plasma levels of *N*-acetyl-neuraminate, 4-pyridoxate, 4-oxopentanoate, glycine, γ-guanidinobutyrate, *N*-γ-ethylglutamine, allantoin, cytosine, 5-methylcytosine and imidazole-4-acetate were significantly increased in the CKD model compared with the sham-operated group, and were significantly decreased by AST-120 treatment. Therefore, these 10 compounds could be added as uremic compounds that indicate the effect of AST-120 treatment. This study provides useful information not only for identifying the indicators of AST-120, but also for clarifying changes in the metabolic profile by AST-120 treatment in the clinical setting.

## 1. Introduction

Chronic kidney disease (CKD) is a global health problem that carries a substantial risk for cardiovascular morbidity and death [1,2]. With the progression of CKD, various uremic toxins accumulate, subsequently causing renal damage and hypertension [3,4,5]. Because most uremic toxins are excreted into the urine, once uremic toxins accumulate in CKD patients, eliminating them is very difficult because of reduced renal function. So far, the only established therapy to eliminate uremic toxins in CKD patients is AST-120 [6,7]. AST-120 is an oral adsorbent that consists of spherical carbon particles and adsorbs uremic toxins and/or their precursors in the intestines. It is reported that AST-120 inhibits the progression of not only CKD but also cardiovascular diseases [6]. One of the best-known uremic toxins whose plasma level is decreased by AST-120 treatment is indoxyl sulfate [8]. AST-120 decreases the plasma level of indoxyl sulfate by adsorbing the precursor of indoxyl sulfate, *i.e.*, the indole, resulting in the reduction of indoxyl sulfate production in the liver. In addition to indoxyl sulfate, some compounds whose plasma levels are decreased by AST-120 treatment have been reported, such as hippurate, phenyl sulfate, 4-ethylphenyl sulfate, p-cresyl sulfate [9] and advanced glycation end products [10]. However, the target compounds of AST-120 have not been fully elucidated.

To clarify the compounds whose plasma concentrations are changed by AST-120 treatment, we comprehensively quantified and analyzed the plasma levels of over 500 compounds in 5/6 nephrectomized (5/6Nx) rats with or without AST-120 treatment using capillary electrophoresis with mass spectrometry (CE-MS). 

## 2. Materials and Methods

### 2.1. Materials

Oral adsorbent AST-120 (Kremezin^®^) was kindly provided by Kureha Corporation (Tokyo, Japan).

### 2.2. Animals and Treatment

Wistar rats were obtained from Charles River (Kanagawa, Japan). At the age of nine weeks, 5/6 nephrectomy or sham operation was performed as previously reported [11]. Ten weeks after the operation, systolic blood pressure (SBP), body weight (BW), plasma creatinine (Cr) and creatinine clearance (Ccr) were measured, and then 5/6Nx rats were randomized into two groups, a control group (*n* = 13) and the AST-120 group (*n* = 12). The AST-120 group was fed powder chow (CE-2, CLEA Japan, Inc., Tokyo, Japan) containing 8% (w/w) AST-120 for four weeks, whereas the sham-operated (*n* = 3) and control (*n* = 13) groups were fed powder chow alone. After four weeks of treatment, SBP, BW, Cr and Ccr were measured again, the rats were then sacrificed and the plasma was subjected to CE-MS analysis. This animal experiment was approved by the Center for Laboratory Animal Research, Tohoku University.

### 2.3. Blood Pressure and Biochemical Measurement

Systolic blood pressure was measured by the tail cuff method (MK-2000, Muromachi Kikai, Tokyo, Japan) in a conscious state. Plasma creatinine was measured with iSTAT (Abbott Point of Care Inc., Princeton, NJ). Urinary creatinine was enzymatically measured (SRL Inc., Tokyo, Japan). 

### 2.4. CE-MS Measurement for Metabolome Analysis

A comprehensive and quantitative analysis of charged metabolites by CE-MS was performed [12,13,14]. Plasma (50 μL) was immediately plunged into methanol (450 μL) containing internal standards (20 μM each of methionine sulfone (Wako, Osaka Japan) for cations, MES (Dojindo, Kumamoto, Japan) and CSA (D-Camphol-10-sulfonic acid, Wako). Then, de-ionized water (200 μL) and chloroform (500 μL) were added, and the mixture was thoroughly mixed. The solution was centrifuged at 4600× *g *for 5 min at 4 °C, and the upper aqueous layer was centrifugally filtered through a Millipore 5000 Da cutoff filter (Millipore, Billerica, MA) to remove proteins. The filtrate was lyophilized and dissolved in 25 μL of Milli-Q water containing reference compounds (200 μM each of 3-aminopyrrolidine (Sigma Aldrich, St. Louis, MO) and trimesate (Wako) prior to capillary electrophoresis with electrospray ionization time-of-flight mass spectrometry (CE-TOFMS) analysis. All CE-TOFMS experiments were performed using the Agilent CE capillary electrophoresis system (Agilent Technologies, Waldbronn, Germany), the Agilent G3250AA LC/MSD TOF system (Agilent Technologies, Palo Alto, CA), the Agilent 1100 series binary HPLC pump, the G1603A Agilent CE-MS adapter, and the G1607A Agilent CE-ESI-MS sprayer kit. For data acquisition, we used G2201AA Agilent ChemStation software for CE and the Analyst QS for Agilent TOFMS software [14]. 

Cationic metabolites were separated in a fused silica capillary (50 μm i.d. × 100 cm) filled with 1 M formic acid as the electrolyte [15]. A sample solution was injected at 50 mbar for 3 s (3 nL) and 30 kV of voltage was applied. The capillary temperature and the sample tray were set at 20 °C and below 5 °C, respectively. Methanol water (50% v/v) containing 0.1 μM Hexakis (2,2-difluorothoxy) phosphazene was delivered as the sheath liquid at 10 μL/min. ESI-TOFMS was operated in the positive ion mode, and the capillary voltage was set at 4 kV. A flow rate of heated dry nitrogen gas (heater temperature 300 °C) was maintained at 10 psig. In TOFMS, the fragmentor, skimmer and Oct RFV voltages were set at 75, 50, and 125 V, respectively. Automatic recalibration of each acquired spectrum was performed using reference masses of reference standards; ([^13^C isotopic ion of protonated methanol dimer (2MeOH + H)]^+^, *m*/*z *66.0632) and ([Hexakis (2,2-difluorothoxy)phosphazene + H]^+^, *m*/*z *622.0290). Exact mass data were acquired at a rate of 1.5 spectra/s over a 50–1000 *m*/*z *range.

As the reference [16], anionic metabolites were separated in a COSMO(+), chemically coated with a cationic polymer, capillary (50 μm i.d. × 100 cm) (Nacalai Tesque, Kyoto, Japan) filled with 50 mM ammonium acetate solution (pH 8.5) as the electrolyte [17]. A sample solution was injected at 50 mbar for 30 s (30 nL) and −30 kV of voltage was applied. A platinum electrospray ionization spray needle was replaced with the original Agilent stainless steel needle [16]. A 5 mM ammonium acetate in 50% (v/v) methanol-water containing 0.1 μM Hexakis (2,2-difluorothoxy) phosphazene was delivered as the sheath liquid at 10 μL/min. ESI-TOFMS was operated in the negative ion mode, and the capillary voltage was set at 3.5 kV. In TOFMS, the fragmentor, skimmer and Oct RFV voltages were set at 100, 50, and 200 V, respectively. Automatic recalibration of each acquired spectrum was performed using reference masses of reference standards; ([^13^C isotopic ion of deprotonated acetic acid dimer (2CH_3_COOH-H)]^−^, *m*/*z*120.0384) and ([Hexakis(2,2-difluorothoxy)phosphazene + deprotonated acetic acid(CH_3_COOH-H)]^−^, *m*/*z *680.0355). Other conditions were as the same as in cationic metabolite analysis.

### 2.5. Statistics

The data were expressed as means ± SD. Samples below the detection limit were assigned the value of the detection limit and subjected to statistical analysis. However, compounds whose levels were below the detection limit in all samples were excluded for this analysis.

Analysis of variance (ANOVA) was performed with the Benjamini-Hochberg multiple testing correction for the false discovery rate at 0.05. Tukey’s procedure for multiple comparisons was applied as post hoc test. *P* < 0.05 was considered to be significant.

## 3. Results

### 3.1. Biological Profiles and CE-MS Analysis

There were no significant differences in the total amounts of food intake (data not shown), SBP, BW, Cr and Ccr between the control and AST-120 groups throughout the experiment (Table 1). An AST-120 rat died of uremia at three weeks. SBP and Cr were significantly lower and Ccr was significantly higher in the sham-operated group than in both the control and AST-120 groups at four weeks of treatment.

Using CE-MS analysis, 220 anionic and 300 cationic compounds whose molecular weights ranged from 71.1 to 784.2 *m/z* were analyzed and quantified. Among them, 146 compounds (61 anions and 85 cations) were detected (Supplementary Tables S1 and S2). The other compounds (159 anions and 215 cations) were below the detection limit in all samples and were excluded for the analysis.

**Table 1:** Parameters before and four weeks after administration of AST-120 to chronic kidney disease (CKD) rats. ** *p* < 0.01 *vs.* control at four weeks.

	Sham (*n* = 3)	Control (*n* = 13)		AST-120 (*n* = 11)	
	4 weeks	0 week	4 weeks	0 week	4 weeks
BW (g)	493 ± 15	452 ± 22	490 ± 19	462 ± 17	486 ± 43
SBP (mmHg)	124 ± 7 **	173 ± 20	177 ± 21	169 ± 23	169 ± 24
Cr (mg/dL)	0.60 ± 0.10 **	1.03 ± 0.12	1.06 ± 0.26	1.04 ± 0.18	0.98 ± 0.26
Ccr (mL/min)	2.09 ± 0.29 **	1.06 ± 0.26	1.29 ± 0.38	1.11 ± 0.28	1.43 ± 0.4

### 3.2. Anions

**Figure 1:**
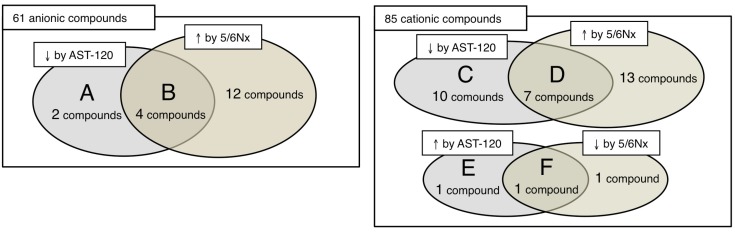
Venn diagram of 61 anionic and 85 cationic compounds evaluated in this study. (**A**) indoxyl sulfate and o-hydroxybezoate. (**B**) 4-oxopentanoate, hippurate, *N*-acetylneuraminate and 4-pyridoxate. (**C**) anthranilate, glycerophosphorylcholine, nicotinamide, glutamine, asparagine, dihydrouracil, glutamate, creatine, γ-butyrobetaine and 1-methylnicotinamide. (**D**) glycine, γ-guanidinobutyrate, *N*-γ-ethylglutamine, allantoin, cytosine, 5-methylcytosine and imidazole-4-acetate. (**E**) Trimethylamine *N*-oxide. (**F**) Tryptophan. Groups B and D may represent uremic compounds that indicate the effects of AST-120.

**Figure 2:**
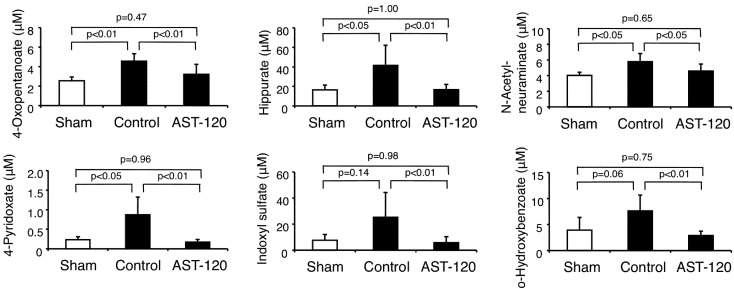
The six anionic compounds that were significantly decreased by AST-120 treatment compared to control.

As shown in Figure 1 and Figure 2, the plasma concentrations of six anionic compounds, 4-oxopentanoate, hippurate, *N*-acetylneuraminate, 4-pyridoxate, indoxyl sulfate and o-hydroxybenzoate, were significantly decreased in the AST-120 group compared to those of the control (A + B group in Figure 1). Four compounds, 4-oxopentanoate, *N*-acetylneuraminate, 4-pyridoxate and o-hydroxybenzoate, were newly indentified as compounds that were significantly decreased by AST-120 treatment*.* The plasma levels of 4-oxopentanoate, hippurate, N-acetylneuraminate and 4-pyridoxate were significantly increased in the control compared with the sham-operated group and significantly decreased by the AST-120 treatment (B group in Figure 1).

### 3.3. Cations

**Figure 3:**
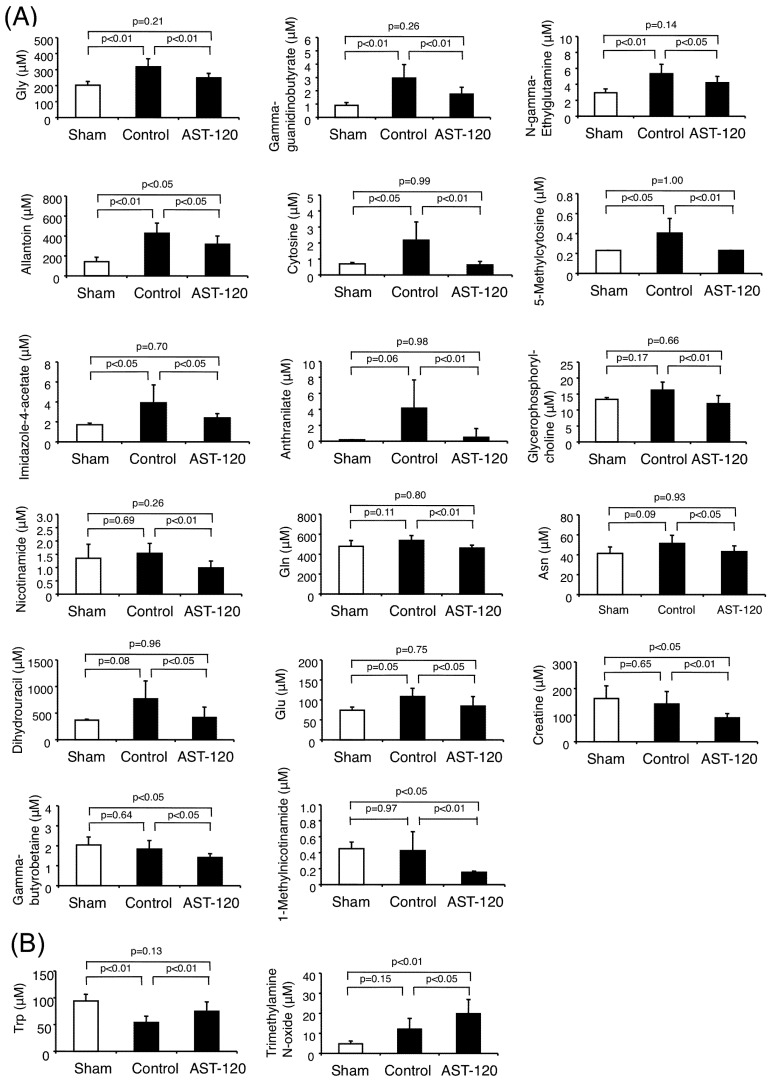
The 19 cationic compounds that were significantly decreased or increased by AST-120 treatment compared to control. (**A**) Compounds that were significantly decreased by AST-120. (**B**) Compounds that were significantly increased by AST-120.

As shown in Figure 1 and Figure 3A, the plasma concentrations of 17 cationic compounds, glycine, γ-guanidinobutyrate, *N*-γ-ethylglutamine, allantoin, cytosine, 5-methylcytosine, imidazole-4-acetate, anthranilate, glycerophosphorylcholine, nicotinamide, glutamine, asparagine, dihydrouracil, glutamate, creatine, γ-butyrobetaine and 1-methylnicotinamide, were significantly decreased in the AST-120 group compared with those in the control (C + D group in Figure 1). All of them are newly identified as compounds that were significantly decreased by AST-120 treatment. The plasma levels of glycine, γ-guanidinobutyrate, *N*-γ-ethylglutamine, allantoin, cytosine, 5-methylcytosine and imidazole-4-acetate were significantly increased in the control group compared with the sham-operated group and significantly decreased by AST-120 treatment (D group in Figure 1).

On the other hand, as shown in Figure 3B, the plasma concentrations of tryptophan and trimethylamine *N*-oxide were significantly increased in the AST-120 group compared to those in the control group (E + F group in Figure 1). Both of them are newly identified as compounds that were significantly increased by AST-120 treatment. The plasma tryptophan concentration was significantly decreased by 5/6Nx and increased to the level of the sham-operated group by AST-120 treatment (F group in Figure 1).

## 4. Discussion

In this study, using the CE-MS method [11,12,13,14,18], we comprehensively analyzed over 500 compounds and quantified 146 compounds (Supplementary Tables S1 and S2). We identified 25 compounds (6 anions and 19 cations) whose plasma concentrations were significantly changed by AST-120 treatment in 5/6Nx rats (the results are summarized in Figure 1 and Table 2). Among them, the plasma levels of 11 compounds were significantly increased in the 5/6Nx rats compared with the sham-operated rats, suggesting that they were uremic compounds whose plasma concentrations were significantly decreased by AST-120 treatment (B and D groups in Figure 1). It has been reported that 4-oxopentanoate, hippurate, allantoin and cytosine also accumulate in CKD patients [13,19,20], suggesting that these four compounds may be common uremic compounds for both rats and human. On the other hand, among the compounds whose plasma concentrations were not changed by 5/6 nephrectomy in this experiment, some have been reported to be accumulated in 5/6Nx rats or CKD patients (Table 2). For example, although it is well known that indoxyl sulfate accumulates in both 5/6Nx rats and CKD patients [8,19], there was no significant difference in the plasma indoxyl sulfate concentration between the sham-operated and control groups in this study. Probably, this was because of the small number (*n* = 3) of sham-operated rats. Other than indoxyl sulfate, asparagine, creatine and γ-butyrobetaine have been reported to be accumulated in CKD patients [13,19]. Therefore, these compounds might be included among the uremic compounds that are indicators of the effects of AST-120. On the other hand, *N*-acetylneuraminate, 4-pyridoxate, *N*-γ-ethylglutamine, 5-methylcytosine and imidazole-4-acetate were not detected in CKD patients in our previous study [13]. Therefore, they may not be uremic compounds in humans. In addition to these, o-hydroxybenzoate, anthranilate, nicotinamide and dihydrouracil were not detected in CKD patients. These discrepancies between 5/6Nx rats and CKD patients may reflect differences in their metabolic profiles. Further studies are needed to clarify the differences in the uremic compounds and the effect of AST-120 between rats and human. 

The plasma levels of creatine, γ-butyrobetaine and 1-methylnicotinamide were significantly lower in the AST-120 group than in the sham-operated group, which suggests that AST-120 may cause a shortage of these compounds. Among them, 1-methylnicotinamide is known as the major metabolite of nicotinamide. Because the plasma level of nicotinamide was also decreased by AST-120, the decrease of 1-methylnicotinamide may be caused by the decrease of nicotinamide. It has been reported that 1-methylnicotinamide and nicotinamide exert anti-inflammatory effects *in vivo *[21], and it has been also reported that 1-methylnicotinamide has anti-thrombotic activity [22] and anti-diabetic effects [23]. Therefore, the decreases of both compounds may have a bad influence on the immune system, cardiovascular system or glucose metabolism in rats. However, nicotinamide was not detected in human serum in our previous study [13]. This may be a result of differences in the profile of nicotinamide metabolism or distribution in the body between rats and human. Further studies are needed to clarify the effect of AST-120 on such compounds in human.

Although AST-120 is an adsorbent, it is unknown whether these compounds are directly adsorbed by AST-120. It is well known that the plasma indoxyl sulfate concentration is decreased by AST-120 treatment because, not indoxyl sulfate itself, but its precursor indole is adsorbed by AST-120 in the intestines. Plasma hippurate concentration is also decreased by AST-120 through adsorbing its precursor benzoic acid [9]. By the same mechanism as indoxyl sulfate and hippurate, the plasma concentration of one metabolite can be decreased if AST-120 adsorbs its precursor and prevents it from being absorbed into the body. In addition, theoretically, compounds that undergo enterohepatic circulation could be adsorbed directly by AST-120. However, so far, little is known about the metabolism and dynamics of the compounds identified in our present study. Therefore the mechanism(s) by which plasma levels of these compounds were lowered by AST-120 still remains largely unknown.

On the other hand, the plasma levels of tryptophan and trimethylamine N-oxide were increased by AST-120 treatment. These increases cannot be explained by the adsorbing effect of AST-120. It has been reported that the plasma tryptophan level is decreased in CKD patients and 5/6Nx rats [13,24]. Because Cr and Ccr did not differ between the control and AST-120 groups, it is suggested that AST-120 recovered the decline of the plasma tryptophan level in the CKD model by unknown mechanism(s) unrelated to renal function. In contrast to tryptophan, it has previously been reported that trimethylamine *N*-oxide accumulates in CKD patients [25]. AST-120 further increased the plasma level of trimethylamine *N*-oxide in 5/6Nx rats. Recently it has been reported that trimethylamine *N*-oxide is formed from dietary phosphatidylcholine through gut-flora-dependent metabolism and promotes atherosclerosis [26]. Therefore the increased level of trimethylamine *N*-oxide may be an adverse effect of AST-120 treatment. In addition, AST-120 may have change the gut-flora composition by its adsorption mechanism in 5/6Nx rats. Further study is needed to clarify the effect of AST-120 on plasma trimethylamine N-oxide level in CKD patients.

Previously, Kikuchi *et al.* reported that indoxyl sulfate, hippurate, phenyl sulfate, 4-ethylphenyl sulfate and *p*-cresyl sulfate were identified as the indicators of the effect of AST-120 in 5/6Nx rats by liquid chromatography/tandem mass spectrometry [9]. Although there were differences in the administration period (three days in Kikuchi *et al.*’s and four weeks in ours), our experimental model was similar to that of Kikuchi *et al.* As same as the result of Kikuchi *et al.*, we also identified indoxyl sulfate and hippurate as the indicators of the effect of AST-120. However, the other compounds, *i.e.*, phenyl sulfate, 4-ethylphenyl sulfate and *p*-cresyl sulfate, were not included in the measurement list in our CE-MS method. Therefore, it is unknown whether the same results were obtained for these compounds in our experimental model. The results of Kikuchi *et al.*, and ours were summarized in Table 2.

**Table 2:** Changes in plasma concentration of the identified compounds in 5/6Nx rats or CKD patients. N.S. indicates that the compound concentration was not significantly changed in renal failure rats or human. Not Detected indicates that the compound concentration in plasma was lower than the detection limit of CE-MS. NA indicates that the compound was not assessed in this experiment.

**Anionic compounds**	**In this experiment**	**Reported by Kikuchi *et al.* [9]**	**Reported in human by Toyohara T. *et al.* [13]**	**Others**	**References**
4-Oxopentanoate	↑ by 5/6Nx, ↓ by AST-120		↑ in CKD patients		
Hippurate	↑ by 5/6Nx, ↓ by AST-120	↑ by 5/6Nx, ↓ by AST-120	↑ in CKD patients	↑ in uremia	[19]
*N*-Acetylneuraminate	↑ by 5/6Nx, ↓ by AST-120		Not Detected		
4-Pyridoxate	↑ by 5/6Nx, ↓ by AST-120		Not Detected		
Indoxyl sulfate	N.S. by 5/6Nx, ↓ by AST-120	↑ by 5/6Nx, ↓ by AST-120	↑ in CKD patients	↑ in uremia	[8,19]
o-Hydroxybenzoate	N.S. by 5/6Nx, ↓ by AST-120		Not Detected		
Phenyl sulfate	NA	↑ by 5/6Nx, ↓ by AST-120	NA		
4-Ethylphenyl sulfate	NA	↑ by 5/6Nx, ↓ by AST-120	NA		
*p*-Cresyl sulfate	NA	↑ by 5/6Nx, ↓ by AST-120	NA		
**Cationic compounds**	**In this experiment**	**Repoted by Kikuchi *et al.* [9]**	**Reported in human by Toyohara T. *et al.* [13]**	**Others**	**References**
Gly	↑ by 5/6Nx, ↓ by AST-120		N.S.		
g-Guanidinobutyrate	↑ by 5/6Nx, ↓ by AST-120		N.S.	↑ in uremia	[19]
*N*-g-Ethylglutamine	↑ by 5/6Nx, ↓ by AST-120		Not Detected		
Allantoin	↑ by 5/6Nx, ↓ by AST-120		↑ in CKD patients	↑ in CKD patients	[20]
Cytosine	↑ by 5/6Nx, ↓ by AST-120		↑ in CKD patients		
5-Methylcytosine	↑ by 5/6Nx, ↓ by AST-120		Not Detected		
Imidazole-4-acetate	↑ by 5/6Nx, ↓ by AST-120		Not Detected		
Anthranilate	N.S. by 5/6Nx, ↓ by AST-120		Not Detected		
Glycerophosphorylcholine	N.S. by 5/6Nx, ↓ by AST-120		N.S.		
Nicotinamide	N.S. by 5/6Nx, ↓ by AST-120		Not Detected		
Gln	N.S. by 5/6Nx, ↓ by AST-120		↓ in CKD patients		
Asn	N.S. by 5/6Nx, ↓ by AST-120		↑ in CKD patients		
Dihydrouracil	N.S. by 5/6Nx, ↓ by AST-120		Not Detected		
Glu	N.S. by 5/6Nx, ↓ by AST-120		N.S.		
Creatine	N.S. by 5/6Nx, ↓ by AST-120		N.S.	↑ in uremia	[19]
g-Butyrobetaine	N.S. by 5/6Nx, ↓ by AST-120		↑ in CKD patients		
1-Methylnicotinamide	N.S. by 5/6Nx, ↓ by AST-120		N.S.		
Trp	↓ by 5/6Nx, ↑ by AST-120		↓ in CKD patients	↓ in uremic rats	[24]
Trimethylamine *N*-oxide	N.S. by 5/6Nx, ↑ by AST-120		↑ in CKD patients	↑ in CKD patients	[25]

## 5. Conclusion

We newly identified 4 anions and 19 cations whose plasma levels are changed by AST-120 treatment in 5/6Nx rats. This study provides useful information, not only for identifying the indicators of AST-120, but also for clarifying changes in the metabolic profile by AST-120 treatment in the clinical setting.

## Supplementary Files

Supplementary File 1: Supplementary File (PDF, 2860 KB)
