# Supplementary material for: A Metabolomic Approach to Clarifying the Effect of AST-120 on 5/6 Nephrectomized Rats by Capillary Electrophoresis with Mass Spectrometry (CE-MS)
Source: Toxins (Basel). 2012 Nov 14;4(11):1309–22. doi: 10.3390/toxins4111309 (PMC3509710; doi:10.3390/toxins4111309)
Supplement: Supplementary File 1: — Supplementary File (PDF, 2860 KB) [file toxins-04-01309-s001.pdf]

# Supplementary Information

**Table S1.** Plasma concentrations of all the anionic compounds detected in this experiment. The data were expressed as means ± SD. S, Sham; C, Control; K, AST-120 (Kremezin®). P values from Tukey's test were shown for the compounds which were significant by ANOVA with Benjamini-Hochberg correction.

| Compound name | Sham (μM) (n=3) | Control (μM) (n=13) | AST-120 (μM) (n=11) | P value (Tukey's multiple comparison procedure) | | |
|---|---|---|---|---|---|---|
| | | | | S vs C | S vs K | C vs K |
| Butanoate | 20.00 ± 5.29 | 8.18 ± 2.21 | 7.33 ± 1.71 | <0.0001 | <0.0001 | 0.6783 |
| 4-Pyridoxate | 0.23 ± 0.08 | 0.87 ± 0.46 | 0.17 ± 0.07 | 0.0149 | 0.9571 | <0.0001 |
| o-Hydroxybenzoate | 3.90 ± 2.46 | 7.60 ± 3.06 | 2.77 ± 1.00 | 0.0562 | 0.7474 | 0.0001 |
| 4-Oxopentanoate | 2.53 ± 0.40 | 4.56 ± 0.76 | 3.20 ± 1.03 | 0.0034 | 0.4731 | 0.0021 |
| Allantoate | 4.47 ± 0.29 | 14.92 ± 3.55 | 12.08 ± 4.19 | 0.0005 | 0.0111 | 0.1661 |
| Citrate | 69.33 ± 24.01 | 238.46 ± 67.15 | 211.64 ± 52.60 | 0.0004 | 0.003 | 0.515 |
| cis-Aconitate | 3.70 ± 1.31 | 11.97 ± 3.20 | 13.04 ± 3.66 | 0.0018 | 0.0006 | 0.712 |
| Malonate | 4.43 ± 1.21 | 6.70 ± 1.07 | 7.41 ± 0.99 | 0.0069 | 0.0006 | 0.2454 |
| Hippurate | 16.33 ± 5.03 | 41.46 ± 20.64 | 16.55 ± 5.53 | 0.0403 | 0.9997 | 0.0014 |
| Isocitrate | 3.27 ± 0.78 | 11.87 ± 3.32 | 10.44 ± 3.57 | 0.0012 | 0.0075 | 0.5474 |
| N-Acetylneuraminate | 4.03 ± 0.40 | 5.78 ± 1.04 | 4.58 ± 0.90 | 0.0208 | 0.6503 | 0.0127 |
| N-Acetylaspartate | 0.63 ± 0.29 | 2.36 ± 0.77 | 2.12 ± 0.73 | 0.003 | 0.012 | 0.6964 |
| Indoxyl sulfate | 7.57 ± 4.50 | 25.18 ± 19.13 | 5.72 ± 4.75 | 0.1403 | 0.9774 | 0.0063 |
| Glutarate | 10.57 ± 1.69 | 26.77 ± 6.87 | 26.45 ± 9.38 | 0.0092 | 0.012 | 0.9946 |
| Saccharate | 0.78 ± 0.00 | 2.10 ± 0.74 | 2.39 ± 0.85 | 0.0303 | 0.0086 | 0.6204 |
| Isethionate | 2.63 ± 0.25 | 4.64 ± 1.82 | 6.53 ± 2.47 | 0.2979 | 0.0202 | 0.0839 |
| Homovanillate | 2.10 ± 0.30 | 6.57 ± 2.65 | 5.70 ± 1.80 | 0.0112 | 0.0489 | 0.6069 |
| Glucuronate+Galacturonate | 3.63 ± 0.85 | 14.28 ± 7.09 | 10.14 ± 4.17 | | | |
| 3-Phenylpropionate | 7.17 ± 1.80 | 9.92 ± 2.26 | 11.25 ± 2.19 | | | |
| Cholate | 4.23 ± 3.44 | 10.10 ± 4.91 | 4.92 ± 4.99 | | | |
| 5-Oxoproline | 40.67 ± 7.64 | 53.38 ± 10.51 | 46.27 ± 5.20 | | | |
| Asp | 6.00 ± 0.35 | 12.35 ± 5.06 | 9.16 ± 3.12 | | | |
| Phenaceturate | 1.30 ± 0.00 | 4.72 ± 4.32 | 1.64 ± 0.69 | | | |
| 2-Hydroxyisobutyrate | 6.53 ± 1.55 | 14.92 ± 5.83 | 13.87 ± 4.18 | | | |
| Azelate | 0.87 ± 0.07 | 1.22 ± 0.33 | 0.97 ± 0.20 | | | |
| Pantothenate | 2.20 ± 0.20 | 3.54 ± 1.10 | 2.76 ± 0.83 | | | |
| 2-Hydroxy-4-methylpentanoate | 0.56 ± 0.00 | 2.84 ± 2.29 | 3.71 ± 1.94 | | | |
| Malate | 16.67 ± 5.86 | 29.92 ± 9.66 | 28.55 ± 8.72 | | | |
| N-Acetylglutamate | 0.43 ± 0.00 | 0.75 ± 0.25 | 0.67 ± 0.21 | | | |
| Gluconate | 8.40 ± 2.29 | 16.54 ± 5.01 | 16.85 ± 7.32 | | | |
| 2-Oxoisopentanoate | 14.67 ± 1.53 | 10.05 ± 4.00 | 10.24 ± 2.41 | | | |
| Pimelate | 0.93 ± 0.04 | 1.78 ± 0.59 | 1.70 ± 0.64 | | | |
| Benzoate | 54.67 ± 19.66 | 38.85 ± 9.86 | 41.45 ± 11.99 | | | |
| Fumarate | 3.50 ± 1.05 | 6.02 ± 2.16 | 5.63 ± 1.75 | | | |
| 3-Hydroxybutyrate | 242.00 ± 23.52 | 318.00 ± 116.97 | 250.00 ± 51.66 | | | |
| Threonate | 14.67 ± 1.15 | 13.31 ± 1.97 | 15.54 ± 3.58 | | | |
| 2-Hydroxybutyrate | 8.13 ± 2.53 | 5.16 ± 1.18 | 6.98 ± 4.05 | | | |
| 3-Phenyllactate | 1.70 ± 0.00 | 5.05 ± 4.20 | 3.22 ± 1.37 | | | |
| 2-Hydroxypentanoate | 18.67 ± 4.16 | 41.62 ± 22.44 | 43.36 ± 18.63 | | | |
| Glycerate | 29.33 ± 11.55 | 42.00 ± 12.75 | 37.91 ± 7.80 | | | |
| Hexanoate | 3.20 ± 0.53 | 3.05 ± 0.76 | 2.66 ± 0.46 | | | |
| Ru5P | 1.20 ± 0.00 | 2.40 ± 1.78 | 1.66 ± 0.83 | | | |
| Glycerophosphate | 1.60 ± 0.00 | 2.35 ± 0.69 | 2.69 ± 1.38 | | | |
| 3-Methylbutanoate | 5.67 ± 0.98 | 5.38 ± 0.44 | 5.21 ± 0.22 | | | |
| Adipate | 1.13 ± 0.15 | 1.54 ± 0.47 | 1.49 ± 0.41 | | | |
| Laurate | 5.37 ± 0.81 | 6.44 ± 1.43 | 5.97 ± 1.23 | | | |
| Succinate | 16.33 ± 4.51 | 18.85 ± 4.95 | 19.64 ± 3.64 | | | |
| Pelargonate | 2.93 ± 0.21 | 3.29 ± 0.58 | 3.14 ± 0.51 | | | |
| Cysteine S-sulfate | 2.07 ± 0.25 | 3.16 ± 2.25 | 2.49 ± 1.44 | | | |
| 4-Methyl-2-oxopentanoate | 34.00 ± 6.08 | 25.69 ± 16.12 | 25.45 ± 7.05 | | | |
| 2-Oxoglutarate | 26.33 ± 8.14 | 35.92 ± 19.68 | 31.91 ± 8.90 | | | |
| Glycolate | 28.00 ± 1.73 | 27.54 ± 2.54 | 25.82 ± 6.65 | | | |
| Octanoate | 1.83 ± 0.51 | 2.05 ± 0.52 | 1.90 ± 0.31 | | | |
| Citraconate | 0.22 ± 0.00 | 0.26 ± 0.10 | 0.33 ± 0.30 | | | |
| Sebacate | 0.23 ± 0.05 | 0.26 ± 0.07 | 0.26 ± 0.05 | | | |
| trans-Aconitate | 2.86 ± 2.98 | 2.59 ± 1.34 | 3.06 ± 1.23 | | | |
| 4-Acetylbutyrate | 0.90 ± 0.87 | 1.26 ± 0.82 | 1.27 ± 0.70 | | | |
| Lactate | 2632.33 ± 639.88 | 2864.15 ± 1463.17 | 2526.82 ± 535.87 | | | |
| Citramalate | 0.62 ± 0.28 | 0.59 ± 0.25 | 0.67 ± 0.33 | | | |
| Propionate | 14.33 ± 10.10 | 13.73 ± 5.34 | 12.80 ± 5.67 | | | |
| Pyruvate | 99.00 ± 26.15 | 107.54 ± 67.44 | 103.27 ± 30.30 | | | |

**Table S2.** Plasma concentrations of all the cationic compounds detected in this experiment. The data were expressed as means ± SD. S, Sham; C, Control; K, AST-120 (Kremezin®). P values from Tukey's test were shown for the compounds which were significant by ANOVA with Benjamini-Hochberg correction.

| Compound name | Sham (μM) (n=3) | Control (μM) (n=13) | AST-120 (μM) (n=11) | P value (Tukey's multiple comparison procedure) | | |
|---|---|---|---|---|---|---|
| | | | | S vs C | S vs K | C vs K |
| Gly | 202.33 ± 23.63 | 317.08 ± 50.97 | 248.73 ± 27.08 | 0.0005 | 0.2068 | 0.0011 |
| Allantoin | 141.33 ± 44.16 | 426.54 ± 101.43 | 316.55 ± 83.39 | 0.0001 | 0.0176 | 0.0179 |
| N,N-Dimethylglycine | 7.20 ± 0.17 | 16.54 ± 3.04 | 15.45 ± 3.08 | 0.0001 | 0.0007 | 0.6436 |
| Trp | 94.00 ± 12.49 | 53.92 ± 11.87 | 74.91 ± 17.44 | 0.0007 | 0.1286 | 0.0047 |
| Cytosine | 0.69 ± 0.09 | 2.17 ± 1.15 | 0.63 ± 0.22 | 0.0259 | 0.9927 | 0.0004 |
| gamma-Guanidinobutyrate | 0.90 ± 0.21 | 2.95 ± 1.02 | 1.75 ± 0.51 | 0.0015 | 0.2563 | 0.0033 |
| Trimethylamine N-oxide | 4.77 ± 1.37 | 12.11 ± 5.33 | 19.82 ± 7.05 | 0.1507 | 0.0019 | 0.0109 |
| 5-Methylcytosine | 0.23 ± 0.00 | 0.40 ± 0.15 | 0.23 ± 0.00 | 0.0373 | 1 | 0.0011 |
| Gln | 478.33 ± 57.50 | 536.08 ± 49.51 | 460.55 ± 29.34 | 0.1131 | 0.8034 | 0.0007 |
| Glycerophosphorylcholine | 13.33 ± 0.58 | 16.23 ± 2.49 | 11.96 ± 2.56 | 0.1697 | 0.6649 | 0.0007 |
| Hydroxyproline | 31.33 ± 2.89 | 49.23 ± 6.13 | 45.27 ± 7.28 | 0.0006 | 0.0078 | 0.3092 |
| N-gamma-Ethylglutamine | 2.93 ± 0.50 | 5.33 ± 1.19 | 4.20 ± 0.79 | 0.0026 | 0.1443 | 0.0271 |
| 1-Methylnicotinamide | 0.45 ± 0.08 | 0.42 ± 0.24 | 0.15 ± 0.02 | 0.9691 | 0.036 | 0.0022 |
| Creatine | 163.00 ± 47.15 | 142.00 ± 46.51 | 90.09 ± 15.69 | 0.6541 | 0.0156 | 0.0061 |
| Homoarginine ; N6,N6,N6-Trimethyllysine | 1.20 ± 0.10 | 2.64 ± 0.58 | 3.03 ± 0.91 | 0.012 | 0.0018 | 0.3955 |
| Citrulline | 57.67 ± 18.15 | 176.15 ± 40.80 | 169.55 ± 59.99 | 0.0024 | 0.0046 | 0.9412 |
| Nicotinamide | 1.35 ± 0.53 | 1.53 ± 0.37 | 0.99 ± 0.25 | 0.6886 | 0.2646 | 0.0022 |
| SDMA | 0.46 ± 0.08 | 0.80 ± 0.12 | 0.82 ± 0.19 | 0.004 | 0.003 | 0.9565 |
| ADMA | 0.18 ± 0.01 | 0.44 ± 0.07 | 0.45 ± 0.16 | 0.005 | 0.0042 | 0.978 |
| Anthranilate | 0.17 ± 0.00 | 4.15 ± 3.54 | 0.50 ± 1.09 | 0.0627 | 0.9793 | 0.0061 |
| Ala | 331.00 ± 43.31 | 499.54 ± 75.89 | 462.45 ± 70.42 | 0.0032 | 0.0245 | 0.4268 |
| gamma-Butyrobetaine | 2.03 ± 0.40 | 1.83 ± 0.43 | 1.41 ± 0.19 | 0.6436 | 0.0298 | 0.0189 |
| Dihydrouracil | 368.33 ± 16.17 | 765.23 ± 338.80 | 417.73 ± 194.78 | 0.0766 | 0.9577 | 0.0121 |
| Imidazole-4-acetate | 1.70 ± 0.17 | 3.89 ± 1.80 | 2.39 ± 0.44 | 0.0382 | 0.6986 | 0.0254 |
| GABA | 1.07 ± 0.14 | 0.81 ± 0.13 | 0.71 ± 0.21 | 0.0515 | 0.0081 | 0.3724 |
| Creatinine | 25.00 ± 1.73 | 74.31 ± 20.74 | 73.09 ± 30.70 | 0.0124 | 0.0167 | 0.992 |
| Glu | 74.33 ± 7.64 | 108.31 ± 20.86 | 84.45 ± 24.09 | 0.0539 | 0.7535 | 0.0322 |
| Asn | 41.33 ± 6.43 | 51.31 ± 8.13 | 43.00 ± 5.88 | 0.0947 | 0.9317 | 0.0236 |
| Thr | 196.00 ± 47.15 | 262.15 ± 45.14 | 222.36 ± 30.38 | | | |
| Spermidine | 0.10 ± 0.00 | 0.28 ± 0.15 | 0.16 ± 0.07 | | | |
| 5-Methyl-2'-deoxycytidine | 0.87 ± 0.14 | 1.23 ± 0.23 | 1.14 ± 0.13 | | | |
| 3-Methylhistidine | 19.33 ± 3.21 | 42.69 ± 6.34 | 45.27 ± 19.81 | | | |
| 1-Methyladenosine | 0.27 ± 0.10 | 0.16 ± 0.07 | 0.15 ± 0.05 | | | |
| alpha-Aminoadipate | 1.43 ± 0.15 | 2.37 ± 0.56 | 2.26 ± 0.49 | | | |
| Pipecolate | 3.23 ± 1.10 | 11.92 ± 2.94 | 13.54 ± 8.00 | | | |
| Pro | 187.00 ± 38.57 | 236.15 ± 37.96 | 241.91 ± 21.09 | | | |
| 4-(beta-Acetylaminoethyl)imidazole | 0.22 ± 0.12 | 0.18 ± 0.09 | 0.12 ± 0.05 | | | |
| N-Acetylglucosamine | 4.77 ± 0.15 | 6.72 ± 1.13 | 6.08 ± 1.52 | | | |
| Glucosamine | 0.61 ± 0.04 | 0.59 ± 0.18 | 0.41 ± 0.24 | | | |
| Taurine | 145.00 ± 57.58 | 276.69 ± 88.64 | 248.82 ± 87.64 | | | |
| Lys | 343.00 ± 38.31 | 466.92 ± 101.17 | 457.64 ± 64.52 | | | |
| Tyramine | 0.20 ± 0.08 | 0.43 ± 0.18 | 0.44 ± 0.17 | | | |
| Asp | 7.50 ± 1.00 | 14.35 ± 4.92 | 12.01 ± 5.34 | | | |
| Triethanolamine | 1.97 ± 0.29 | 3.88 ± 1.30 | 3.92 ± 1.75 | | | |
| Gly-Leu | 0.40 ± 0.00 | 0.73 ± 0.31 | 0.59 ± 0.23 | | | |
| Putrescine(1,4-Butanediamine) | 0.68 ± 0.07 | 0.58 ± 0.21 | 0.48 ± 0.11 | | | |
| Glutathione(ox); Glutathione(ox) divalent | 0.55 ± 0.00 | 1.34 ± 0.88 | 0.91 ± 0.59 | | | |
| 5-Hydroxylysine | 1.47 ± 0.15 | 1.65 ± 0.62 | 2.04 ± 0.55 | | | |
| Cystathionine | 1.20 ± 0.00 | 1.86 ± 0.55 | 1.72 ± 0.58 | | | |
| Sarcosine | 6.17 ± 0.90 | 7.95 ± 1.87 | 8.07 ± 1.26 | | | |
| o-Acetylcarnitine | 8.13 ± 1.35 | 8.74 ± 2.59 | 7.03 ± 1.91 | | | |
| Cytidine | 3.13 ± 0.68 | 3.88 ± 1.03 | 3.29 ± 0.80 | | | |
| Betonicine | 0.37 ± 0.09 | 0.56 ± 0.18 | 0.49 ± 0.16 | | | |
| Phe | 58.67 ± 4.04 | 63.77 ± 10.69 | 68.00 ± 6.74 | | | |
| Guanidoacetate | 5.47 ± 0.95 | 5.68 ± 1.33 | 4.88 ± 0.85 | | | |
| beta-Ala | 3.27 ± 0.31 | 2.91 ± 0.45 | 2.75 ± 0.49 | | | |
| 7-Methylguanine | 0.17 ± 0.00 | 0.28 ± 0.11 | 0.26 ± 0.10 | | | |
| Hypotaurine | 3.80 ± 0.72 | 3.75 ± 0.88 | 3.28 ± 0.49 | | | |
| N-epsilon-Acetyllysine | 0.79 ± 0.13 | 1.02 ± 0.31 | 1.04 ± 0.13 | | | |
| Ectoine | 1.50 ± 0.40 | 4.55 ± 3.77 | 4.09 ± 1.49 | | | |
| Azetidine-2-carboxylate | 0.61 ± 0.00 | 0.85 ± 0.25 | 0.86 ± 0.26 | | | |
| Kynurenine | 1.30 ± 0.20 | 1.64 ± 0.35 | 1.66 ± 0.39 | | | |
| Arg | 137.00 ± 23.64 | 275.85 ± 164.02 | 255.09 ± 119.46 | | | |
| Ser | 163.00 ± 30.32 | 183.23 ± 32.17 | 164.82 ± 34.44 | | | |
| Guanidinosuccinate | 0.39 ± 0.23 | 0.54 ± 0.23 | 0.71 ± 0.56 | | | |
| Ornithine | 52.00 ± 21.93 | 75.54 ± 17.28 | 71.55 ± 36.26 | | | |
| Anserine | 1.62 ± 0.96 | 1.08 ± 0.78 | 1.77 ± 1.81 | | | |
| 2'-Deoxycytidine | 23.33 ± 4.04 | 20.69 ± 3.66 | 20.55 ± 3.01 | | | |
| Carnitine | 64.33 ± 8.39 | 56.00 ± 12.48 | 59.73 ± 10.18 | | | |
| Betaine | 144.00 ± 44.54 | 124.46 ± 23.10 | 134.82 ± 29.16 | | | |
| Met | 48.33 ± 5.51 | 59.85 ± 21.20 | 63.27 ± 16.86 | | | |
| Val | 167.67 ± 23.44 | 153.69 ± 22.79 | 163.64 ± 27.19 | | | |
| Leu | 136.00 ± 20.88 | 133.00 ± 33.29 | 147.18 ± 29.31 | | | |
| Carnosine; Carnosine divalent | 0.36 ± 0.06 | 0.37 ± 0.17 | 0.44 ± 0.22 | | | |
| Methionine sulfoxide | 7.23 ± 1.42 | 8.03 ± 1.87 | 7.51 ± 0.81 | | | |
| Uridine | 16.00 ± 0.00 | 22.46 ± 11.00 | 23.36 ± 12.32 | | | |
| His | 64.33 ± 5.51 | 69.92 ± 10.28 | 68.36 ± 8.04 | | | |
| Urocanate | 0.99 ± 0.53 | 2.11 ± 2.00 | 4.96 ± 12.01 | | | |
| 2AB; 2-Aminobutyrate | 4.97 ± 0.61 | 4.90 ± 1.02 | 4.59 ± 0.75 | | | |
| Thiamine | 8.23 ± 1.53 | 9.77 ± 4.36 | 8.63 ± 2.92 | | | |
| Cysteine-glutathione disulphide | 0.89 ± 0.28 | 1.40 ± 1.12 | 1.22 ± 0.92 | | | |
| 5-Oxoproline | 27.00 ± 1.73 | 33.85 ± 8.21 | 31.55 ± 19.79 | | | |
| Tyr | 40.67 ± 4.04 | 46.15 ± 13.92 | 45.09 ± 9.21 | | | |
| Histamine | 0.28 ± 0.10 | 0.35 ± 0.30 | 0.30 ± 0.10 | | | |
| Ile | 68.33 ± 9.50 | 67.54 ± 11.84 | 69.45 ± 13.44 | | | |
